# Association of obesity–metabolic phenotypes with coronary artery disease prevalence: interaction and exploratory mediation analyses of high-sensitivity C-reactive protein

**DOI:** 10.3389/fcvm.2026.1961932

**Published:** 2026-09-16

**Authors:** Chunjing Zhu, Yazhao Sun

**Affiliations:** 1 Department of Endocrinology, Cangzhou People's Hospital, Cangzhou, China; 2 Department of Cardiology, Cangzhou People's Hospital, Cangzhou, China

**Keywords:** coronary artery disease, high-sensitivity c-reactive protein, interaction, mediation analysis, metabolic health, obesity

## Abstract

**Objective:**

To investigate the associations of obesity–metabolic phenotypes with coronary artery disease (CAD) prevalence and to evaluate the interaction between obesity and metabolic unhealthiness and the potential mediating role of high-sensitivity C-reactive protein (hsCRP).

**Methods:**

A total of 860 participants were classified as metabolically healthy non-obese (MHNO), metabolically healthy obesity (MHO), metabolically unhealthy non-obese (MUNO), or metabolically unhealthy obesity (MUO). Associations with CAD prevalence were estimated using modified Poisson regression with robust variance. Interactions between obesity and metabolic unhealthiness were assessed on multiplicative and additive scales. Subgroup and sensitivity analyses were performed, followed by an exploratory mediation analysis of hsCRP.

**Results:**

Among 860 participants, 563 (65.5%) had CAD. After adjustment for age, sex, current smoking, alcohol use, family history of CAD, lipoprotein(a) [Lp(a)], low-density lipoprotein cholesterol (LDL-C), and estimated glomerular filtration rate (eGFR), the prevalence ratios for CAD were 1.51 [95% confidence interval (CI), 1.26–1.81] for MHO, 1.73 (95% CI, 1.48–2.02) for MUNO, and 1.79 (95% CI, 1.53–2.10) for MUO compared with MHNO (all *P* < 0.001). Negative interactions between obesity and metabolic unhealthiness were observed on both the multiplicative scale (interaction ratio, 0.69; 95% CI, 0.56–0.84) and the additive scale [relative excess due to interaction (RERI), −0.44; 95% CI, −0.79 to −0.13]. Results were consistent across sensitivity analyses. Exploratory mediation analysis showed average indirect effects through hsCRP of 1.94, 5.93, and 8.20 percentage points for MHO, MUNO, and MUO, respectively, corresponding to proportions mediated of 9.4%, 19.6%, and 23.6%.

**Conclusions:**

MHO, MUNO, and MUO were all associated with higher CAD prevalence, with stronger associations observed for metabolically unhealthy phenotypes. Obesity and metabolic unhealthiness showed a negative interaction. Exploratory findings indicated an hsCRP-related indirect association, but causal mediation cannot be inferred from this cross-sectional study.

## Introduction

Obesity is closely associated with cardiovascular disease, yet individuals with a similar degree of adiposity may differ substantially in their cardiometabolic profiles. The American Heart Association has emphasized that obesity is linked not only to conventional cardiovascular risk factors, including hypertension, dyslipidemia, and impaired glucose metabolism, but also to atherosclerotic cardiovascular disease itself (1). This heterogeneity has led to increasing interest in obesity–metabolic phenotypes, particularly metabolically healthy obesity (MHO) and metabolically unhealthy obesity (MUO) (2). Recent cohort studies have shown that cardiovascular outcomes vary across obesity–metabolic phenotypes. Filippatos et al. reported higher long-term cardiovascular disease incidence among individuals with MHO and MUO in the ATTICA cohort (3). In a nationwide Chinese cohort, He et al. found that both obesity and metabolic health status, as well as transitions between metabolic states, were associated with incident cardiovascular disease (4). Similar differences across obesity–metabolic phenotypes have also been observed in middle-aged and older Chinese adults (5).

Evidence linking obesity–metabolic phenotypes specifically to coronary atherosclerosis and coronary artery disease (CAD), however, remains less consistent. In the Prospective Multicenter Imaging Study for Evaluation of Chest Pain (PROMISE), Kammerlander et al. found that metabolically unhealthy phenotypes were associated with a more adverse coronary profile, whereas MHO did not differ significantly from metabolically healthy non-obese individuals in severe coronary artery calcification, obstructive CAD, or high-risk plaque (6). By contrast, Kowall et al. reported that MHO was associated with both the prevalence and progression of coronary artery calcification in the Heinz Nixdorf Recall cohort (7). Palatini et al. further showed that cardiovascular outcomes among individuals with MHO may vary according to low-density lipoprotein cholesterol (LDL-C) levels and the definition of metabolic health (8). More recently, Kim et al. demonstrated that apolipoprotein B and LDL-C discordance further differentiated atherosclerotic burden across metabolic phenotypes (9). Despite these findings, data based on clinically indicated coronary angiography remain limited, and the interaction between obesity and metabolic unhealthiness in relation to CAD has not been fully characterized.

Chronic low-grade inflammation may provide an additional link between obesity, metabolic dysfunction, and coronary atherosclerosis. A systematic review and meta-analysis by Su et al. showed that inflammatory marker levels differ across obesity–metabolic phenotypes, with a greater inflammatory burden generally observed in metabolically unhealthy obesity (10). Baek et al. further demonstrated substantial differences in high-sensitivity C-reactive protein (hsCRP) across metabolic obesity phenotypes (11), while proteomic data from Liao et al. implicated inflammatory and immune-regulatory pathways in the biological heterogeneity of these phenotypes (12). In relation to coronary atherosclerosis, Chen et al. recently reported a greater burden of coronary artery calcification among individuals with both metabolic syndrome and elevated hsCRP (13). The 2025 American College of Cardiology Scientific Statement also highlighted the importance of chronic inflammation and hsCRP in atherosclerotic cardiovascular disease (14). Nevertheless, whether hsCRP contributes to the association between obesity–metabolic phenotypes and CAD remains insufficiently understood. Therefore, this study aimed to evaluate the association between obesity–metabolic phenotypes and CAD prevalence, assess the interaction between obesity and metabolic unhealthiness, and explore the potential mediating role of hsCRP.

## Methods

### Study design and participants

This single-center retrospective cross-sectional study included patients who underwent their first coronary angiography at Cangzhou People's Hospital between July 2023 and February 2026. Eligible patients were required to have complete and interpretable coronary angiographic data, as well as sufficient clinical and laboratory information for classification of obesity–metabolic phenotypes and subsequent analyses. Patients with a previous diagnosis of CAD, uninterpretable coronary angiographic findings, or missing key clinical or laboratory data were excluded. A total of 860 participants were included in the final analysis. The study was approved by the Ethics Committee of Cangzhou People's Hospital (approval No. K2025-130-02). Written informed consent permitting the use of clinical data for medical research was obtained from all patients at admission.

### Clinical data collection

Clinical data were retrieved from the electronic medical record system for the hospitalization during which patients underwent their first coronary angiography. Collected information included demographic characteristics, lifestyle factors, medical history, anthropometric measurements, blood pressure, medication use, clinical presentation, and laboratory findings. Specifically, data were obtained on age, sex, current smoking, alcohol use, family history of CAD, height, weight, systolic and diastolic blood pressure, hypertension, diabetes mellitus, use of antihypertensive, glucose-lowering, and statin medications, and presentation with acute coronary syndrome (ACS).

Laboratory measurements included fasting plasma glucose (FPG), glycated hemoglobin (HbA1c), triglycerides (TG), high-density lipoprotein cholesterol (HDL-C), LDL-C, total cholesterol (TC), lipoprotein(a) [Lp(a)], uric acid, creatinine, estimated glomerular filtration rate (eGFR), and hsCRP.

### Definition of obesity–metabolic phenotypes

Body mass index (BMI) was calculated as weight in kilograms divided by height in meters squared. Obesity was defined as a BMI ≥28 kg/m^2^. Metabolic health status was determined according to four metabolic components: blood pressure, glucose metabolism, TG, and HDL-C. Abnormal blood pressure was defined as systolic blood pressure ≥130 mmHg, diastolic blood pressure ≥85 mmHg, a previous diagnosis of hypertension, or current use of antihypertensive medication. Abnormal glucose metabolism was defined as fasting plasma glucose ≥5.6 mmol/L, a previous diagnosis of diabetes mellitus, or current use of glucose-lowering medication. TG ≥1.7 mmol/L was considered abnormal, and low HDL-C was defined as <1.0 mmol/L in men and <1.3 mmol/L in women.

Participants with 0-1 metabolic abnormality were classified as metabolically healthy, whereas those with ≥2 abnormalities were classified as metabolically unhealthy. This definition was based on previous studies of obesity–metabolic phenotypes, in which metabolic health status is generally determined by the presence of multiple cardiometabolic abnormalities rather than a single isolated abnormality (2). Based on obesity and metabolic health status, participants were categorized into four phenotypes: metabolically healthy non-obese (MHNO), MHO, metabolically unhealthy non-obese (MUNO), and MUO.

### Coronary angiography and outcome definition

All participants underwent their first coronary angiography during the index hospitalization. The maximum degree of coronary artery stenosis was recorded based on the angiographic findings. CAD was defined as a maximum coronary stenosis of ≥50%, whereas a stenosis of <50% was classified as non-CAD. The presence of CAD was the primary outcome. In sensitivity analyses, severe CAD was defined as a maximum coronary stenosis of ≥70%.

### Statistical analysis

Normally distributed continuous variables were expressed as mea*n* ± standard deviation and compared across the four groups using one-way analysis of variance. Skewed continuous variables were expressed as median (first quartile, third quartile) and compared using the Kruskal–Wallis test. Categorical variables were presented as *n* (%) and compared using Pearson's *χ*^2^ test.

Using MHNO as the reference group, modified Poisson regression with a heteroskedasticity-consistent type 0 (HC0) robust variance estimator was used to estimate prevalence ratios (PRs) and 95% confidence intervals (CIs) for the association between obesity–metabolic phenotypes and CAD prevalence. Model 1 was unadjusted, whereas Model 2 was adjusted for age, sex, current smoking, alcohol use, family history of CAD, Lp(a), LDL-C, and eGFR. These covariates were selected *a priori* based on their established associations with coronary artery disease and potential roles as cardiovascular risk-related factors. The overall association of obesity–metabolic phenotype with CAD prevalence was assessed using a joint robust Wald test. Logistic regression was additionally performed to estimate odds ratios (ORs). Covariate-adjusted CAD prevalence for each phenotype was obtained by marginal standardization based on the adjusted model, with 95% CIs calculated using the delta method. Interactions between obesity and metabolic unhealthiness were assessed on both multiplicative and additive scales. Multiplicative interaction was evaluated by including an obesity-by-metabolic-unhealthiness interaction term in the modified Poisson model. Additive-scale interaction on the prevalence scale was assessed using the relative excess due to interaction (RERI), attributable proportion due to interaction (AP), and synergy index (S), calculated from adjusted PRs. Their 95% CIs and *P* values were estimated using 1,000 nonparametric bootstrap resamples. Subgroup analyses were conducted according to age, sex, current smoking status, eGFR, Lp(a) level, and family history of CAD. Each subgroup model was adjusted for the prespecified covariates except the corresponding stratification variable, and interaction was assessed using an overall robust Wald test of the phenotype-by-subgroup interaction terms. Subgroup analyses were exploratory, and no adjustment for multiple comparisons was applied.

Sensitivity analyses included redefining severe CAD as maximum coronary artery stenosis ≥70%, modeling age, Lp(a), LDL-C, and eGFR using restricted cubic splines, excluding patients with ACS, and additionally adjusting for statin use. Restricted cubic splines were specified with three knots at the 10th, 50th, and 90th percentiles of each continuous covariate. Adjusted PRs and 95% CIs in all sensitivity analyses were estimated using modified Poisson regression with HC0 robust variance. Exploratory mediation analyses were performed separately for MHO, MUNO, and MUO versus MHNO, with hsCRP as the mediator. The mediator model was fitted using linear regression, and the CAD outcome model was fitted using logistic regression including a phenotype-by-hsCRP interaction term. Both models were adjusted for the prespecified covariates described above. The average indirect effect (ACME), average direct effect (ADE), total effect (TE), and proportion mediated (PM) were estimated. ACME, ADE, and TE were expressed as absolute differences in the predicted probability of CAD. For the mediation effects, 95% CIs and *P* values were obtained using 1,000 quasi-Bayesian Monte Carlo simulations. All statistical analyses were performed using R software (version 4.4.3). All tests were two-sided, and *P* < 0.05 was considered statistically significant.

## Results

### Baseline characteristics

A total of 860 participants were included, of whom 563 (65.5%) had CAD. The MHNO, MHO, MUNO, and MUO groups comprised 260, 145, 265, and 190 participants, respectively. Age and sex differed across the four groups, whereas no significant differences were observed in current smoking, alcohol use, or family history of CAD. Significant between-group differences were also observed in uric acid, creatinine, eGFR, statin use, ACS, and hsCRP.

Median hsCRP levels were 1.40, 1.68, 2.18, and 2.79 mg/L in the MHNO, MHO, MUNO, and MUO groups, respectively (*P* < 0.001). The corresponding crude prevalence of CAD was 41.5%, 64.8%, 77.4%, and 82.1%, respectively (*P* < 0.001; Table 1).

**Table 1.** Baseline characteristics of the study population according to obesity–metabolic phenotypes.

Characteristic	Overall (*N* = 860)	MHNO (*n* = 260)	MHO (*n* = 145)	MUNO (*n* = 265)	MUO (*n* = 190)	*P* value
Demographic and lifestyle characteristics						
Age, years	62.0 ± 7.3	60.1 ± 7.2	60.6 ± 7.7	63.4 ± 7.2	63.7 ± 6.6	<0.001
Male sex, *n* (%)	500 (58.1)	136 (52.3)	70 (48.3)	177 (66.8)	117 (61.6)	<0.001
Current smoking, *n* (%)	348 (40.5)	101 (38.8)	65 (44.8)	110 (41.5)	72 (37.9)	0.558
Alcohol use, *n* (%)	280 (32.6)	84 (32.3)	48 (33.1)	83 (31.3)	65 (34.2)	0.930
Family history of CAD, *n* (%)	151 (17.6)	47 (18.1)	23 (15.9)	40 (15.1)	41 (21.6)	0.312
Anthropometric and blood pressure measures						
Height, cm	166.1 ± 8.1	165.5 ± 8.0	165.2 ± 9.1	167.0 ± 7.8	166.2 ± 7.6	0.107
Weight, kg	74.1 ± 13.0	64.8 ± 7.5	84.1 ± 10.5	68.0 ± 7.7	87.9 ± 9.1	<0.001
BMI, kg/m^2^	26.8 ± 4.0	23.6 ± 1.7	30.7 ± 1.5	24.3 ± 1.7	31.7 ± 1.6	<0.001
Obesity (BMI ≥28 kg/m^2^), *n* (%)	335 (39.0)	0 (0.0)	145 (100.0)	0 (0.0)	190 (100.0)	<0.001
Systolic blood pressure, mmHg	124.9 ± 12.7	120.3 ± 9.8	120.4 ± 10.6	129.3 ± 12.9	128.5 ± 13.9	<0.001
Diastolic blood pressure, mmHg	77.1 ± 7.4	74.6 ± 6.0	75.4 ± 6.6	79.1 ± 7.4	79.0 ± 8.1	<0.001
Hypertension, *n* (%)	261 (30.3)	27 (10.4)	19 (13.1)	119 (44.9)	96 (50.5)	<0.001
Antihypertensive medication, *n* (%)	179 (20.8)	20 (7.7)	11 (7.6)	84 (31.7)	64 (33.7)	<0.001
Glycolipid metabolism and renal function						
FPG, mmol/L	5.28 (4.95, 6.13)	5.08 (4.84, 5.33)	5.04 (4.83, 5.27)	5.84 (5.14, 6.39)	5.96 (5.23, 6.59)	<0.001
HbA1c, %	5.5 (5.2, 6.1)	5.3 (5.1, 5.6)	5.3 (5.1, 5.6)	5.8 (5.4, 6.5)	6.0 (5.5, 6.9)	<0.001
Diabetes mellitus, *n* (%)	179 (20.8)	10 (3.8)	7 (4.8)	79 (29.8)	83 (43.7)	<0.001
Glucose-lowering medication, *n* (%)	113 (13.1)	4 (1.5)	1 (0.7)	57 (21.5)	51 (26.8)	<0.001
TG, mmol/L	1.48 (1.17, 2.20)	1.23 (1.06, 1.46)	1.29 (1.06, 1.48)	1.92 (1.32, 2.36)	2.29 (1.77, 2.70)	<0.001
HDL-C, mmol/L	1.23 ± 0.30	1.37 ± 0.26	1.38 ± 0.26	1.12 ± 0.29	1.06 ± 0.24	<0.001
LDL-C, mmol/L	2.73 ± 0.74	2.70 ± 0.75	2.68 ± 0.68	2.76 ± 0.74	2.77 ± 0.77	0.470
TC, mmol/L	4.73 ± 0.84	4.67 ± 0.80	4.65 ± 0.80	4.74 ± 0.86	4.87 ± 0.87	0.055
Lp(a), mg/L	152.8 (89.9, 273.4)	140.9 (87.3, 258.3)	160.2 (91.0, 276.6)	151.4 (89.6, 266.3)	171.4 (91.1, 305.6)	0.205
Uric acid, μmol/L	352.6 ± 70.7	323.4 ± 63.9	350.3 ± 77.0	347.8 ± 60.4	400.9 ± 63.2	<0.001
Creatinine, μmol/L	82.0 (66.0, 100.0)	73.5 (62.5, 90.0)	81.0 (68.0, 99.0)	83.0 (68.0, 100.0)	88.0 (72.0, 115.0)	<0.001
eGFR, mL/min/1.73 m^2^	80.7 ± 22.6	86.7 ± 22.8	79.2 ± 21.6	80.8 ± 21.8	73.7 ± 21.9	<0.001
Treatment and clinical presentation						
Statin use, *n* (%)	332 (38.6)	73 (28.1)	48 (33.1)	119 (44.9)	92 (48.4)	<0.001
ACS, *n* (%)	220 (25.6)	53 (20.4)	33 (22.8)	77 (29.1)	57 (30.0)	0.048
Inflammatory marker						
hsCRP, mg/L	1.90 (1.29, 2.88)	1.40 (1.05, 1.88)	1.68 (1.25, 2.46)	2.18 (1.46, 2.98)	2.79 (1.94, 3.84)	<0.001
Components of the obesity–metabolic phenotype						
Blood pressure abnormality, *n* (%)	377 (43.8)	43 (16.5)	25 (17.2)	184 (69.4)	125 (65.8)	<0.001
Glucose metabolism abnormality, *n* (%)	376 (43.7)	42 (16.2)	15 (10.3)	178 (67.2)	141 (74.2)	<0.001
TG abnormality, *n* (%)	358 (41.6)	29 (11.2)	16 (11.0)	169 (63.8)	144 (75.8)	<0.001
HDL-C abnormality, *n* (%)	337 (39.2)	30 (11.5)	16 (11.0)	157 (59.2)	134 (70.5)	<0.001
Number of metabolic abnormalities	2 (1, 3)	1 (0, 1)	0 (0, 1)	2 (2, 3)	3 (2, 3)	<0.001
Metabolically unhealthy status, *n* (%)	455 (52.9)	0 (0.0)	0 (0.0)	265 (100.0)	190 (100.0)	<0.001
Coronary angiography and CAD						
Maximum coronary artery stenosis, %	64.0 (30.0, 76.0)	34.5 (20.5, 67.0)	63.0 (29.0, 77.0)	68.0 (52.0, 80.0)	69.0 (57.0, 77.0)	<0.001
CAD, *n* (%)	563 (65.5)	108 (41.5)	94 (64.8)	205 (77.4)	156 (82.1)	<0.001

ACS, acute coronary syndrome; BMI, body mass index; CAD, coronary artery disease; eGFR, estimated glomerular filtration rate; FPG, fasting plasma glucose; HbA1c, glycated hemoglobin; HDL-C, high-density lipoprotein cholesterol; hsCRP, high-sensitivity C-reactive protein; LDL-C, low-density lipoprotein cholesterol; Lp(a), lipoprotein(a); MHNO, metabolically healthy non-obese; MHO, metabolically healthy obesity; MUNO, metabolically unhealthy non-obese; MUO, metabolically unhealthy obesity; TC, total cholesterol; TG, triglycerides.

### Association between obesity–metabolic phenotypes and CAD prevalence

To evaluate the association between obesity–metabolic phenotypes and CAD prevalence, modified Poisson regression was performed using two models. Model 1 was unadjusted, whereas Model 2 was adjusted for age, sex, current smoking, alcohol use, family history of CAD, Lp(a), LDL-C, and eGFR. With the MHNO group as the reference, the PRs for MHO, MUNO, and MUO were 1.56 (95% CI, 1.29–1.88), 1.86 (95% CI, 1.59–2.18), and 1.98 (95% CI, 1.69–2.32), respectively, in Model 1. The corresponding PRs in Model 2 were 1.51 (95% CI, 1.26–1.81), 1.73 (95% CI, 1.48–2.02), and 1.79 (95% CI, 1.53–2.10), respectively, with all *P* < 0.001. The overall association between obesity–metabolic phenotype and CAD prevalence was also statistically significant (*P* < 0.001).

In the logistic regression analysis, the adjusted ORs for MHO, MUNO, and MUO versus MHNO were 2.57 (95% CI, 1.64–4.03), 4.35 (95% CI, 2.90–6.51), and 5.64 (95% CI, 3.51–9.06), respectively, with all *P* < 0.001. After marginal standardization, the adjusted prevalence of CAD was 45.5% (95% CI, 39.7%–51.4%) in the MHNO group, 65.9% (95% CI, 58.6%–73.3%) in the MHO group, 75.7% (95% CI, 70.5%–80.8%) in the MUNO group, and 79.8% (95% CI, 74.0%–85.6%) in the MUO group (Table 2).

**Table 2.** Association between obesity–metabolic phenotypes and CAD prevalence.

Phenotype	*n*	CAD, *n* (%)	Model 1 PR (95% CI)	Model 2 PR (95% CI)	Model 2 OR (95% CI)	Adjusted prevalence, % (95% CI)
MHNO	260	108 (41.5)	1.00 (Reference)	1.00 (Reference)	1.00 (Reference)	45.5 (39.7–51.4)
MHO	145	94 (64.8)	1.56 (1.29–1.88)	1.51 (1.26–1.81)	2.57 (1.64–4.03)	65.9 (58.6–73.3)
MUNO	265	205 (77.4)	1.86 (1.59–2.18)	1.73 (1.48–2.02)	4.35 (2.90–6.51)	75.7 (70.5–80.8)
MUO	190	156 (82.1)	1.98 (1.69–2.32)	1.79 (1.53–2.10)	5.64 (3.51–9.06)	79.8 (74.0–85.6)

Model 1 was unadjusted. Model 2 was adjusted for age, sex, current smoking, alcohol use, family history of CAD, Lp(a), LDL-C, and eGFR. PRs were estimated using modified Poisson regression with HC0 robust variance. ORs were estimated using logistic regression. Adjusted prevalence estimates were obtained by marginal standardization based on Model 2, with 95% CIs calculated using the delta method.

CAD, coronary artery disease; CI, confidence interval; eGFR, estimated glomerular filtration rate; LDL-C, low-density lipoprotein cholesterol; Lp(a), lipoprotein(a); MHNO, metabolically healthy non-obese; MHO, metabolically healthy obesity; MUNO, metabolically unhealthy non-obese; MUO, metabolically unhealthy obesity; OR, odds ratio; PR, prevalence ratio.

### Joint association and interaction between obesity and metabolic unhealthiness

The joint association and interaction between obesity and metabolic unhealthiness with CAD prevalence were further evaluated. Using the MHNO group as the reference, the adjusted PRs were 1.51 (95% CI, 1.26–1.81) for MHO, 1.73 (95% CI, 1.48–2.02) for MUNO, and 1.79 (95% CI, 1.53–2.10) for MUO. On the multiplicative scale, the interaction ratio between obesity and metabolic unhealthiness was 0.69 (95% CI, 0.56–0.84; *P* < 0.001).

On the additive scale, the RERI was −0.44 (95% CI, −0.79 to −0.13; *P* = 0.004), the AP was −0.25 (95% CI, −0.42 to −0.07; *P* = 0.004), and the S was 0.64 (95% CI, 0.52–0.86; *P* = 0.004). Both multiplicative and additive interaction measures indicated a negative interaction (Table 3).

**Table 3.** Joint association and interaction between obesity and metabolic unhealthiness.

Measure	Estimate (95% CI)	*P* value
MHO vs MHNO, adjusted PR	1.51 (1.26–1.81)	<0.001
MUNO vs MHNO, adjusted PR	1.73 (1.48–2.02)	<0.001
MUO vs MHNO, adjusted PR	1.79 (1.53–2.10)	<0.001
Multiplicative interaction ratio	0.69 (0.56–0.84)	<0.001
RERI	−0.44 (−0.79 to −0.13)	0.004
AP	−0.25 (−0.42 to −0.07)	0.004
S	0.64 (0.52–0.86)	0.004

Adjusted PRs and the multiplicative interaction ratio were estimated using modified Poisson regression with HC0 robust variance. RERI, AP, and S were calculated based on the adjusted PRs. Their 95% CIs and P values were estimated using 1,000 nonparametric bootstrap resamples.AP, attributable proportion due to interaction; CI, confidence interval; MHNO, metabolically healthy non-obese; MHO, metabolically healthy obesity; MUNO, metabolically unhealthy non-obese; MUO, metabolically unhealthy obesity; PR, prevalence ratio; RERI, relative excess due to interaction; S, synergy index.

### Subgroup analysis

Subgroup analyses were further performed according to age, sex, current smoking status, eGFR, Lp(a) level, and family history of CAD. The associations of MHO, MUNO, and MUO with CAD prevalence were generally consistent across subgroups. No significant interactions were observed for age, sex, eGFR, or Lp(a), with *P* values for interaction of 0.096, 0.297, 0.141, and 0.690, respectively. The nominal *P* values for interaction were 0.015 for current smoking status and 0.037 for family history of CAD.

In the subgroup with Lp(a) ≥ 500 mg/L, only 83 participants were included, and the 95% CIs for MHO, MUNO, and MUO all crossed 1. In addition, 10 subgroup-by-phenotype cells contained fewer than 10 CAD events or non-events, resulting in less precise estimates (Figure 1).

**Figure 1:**
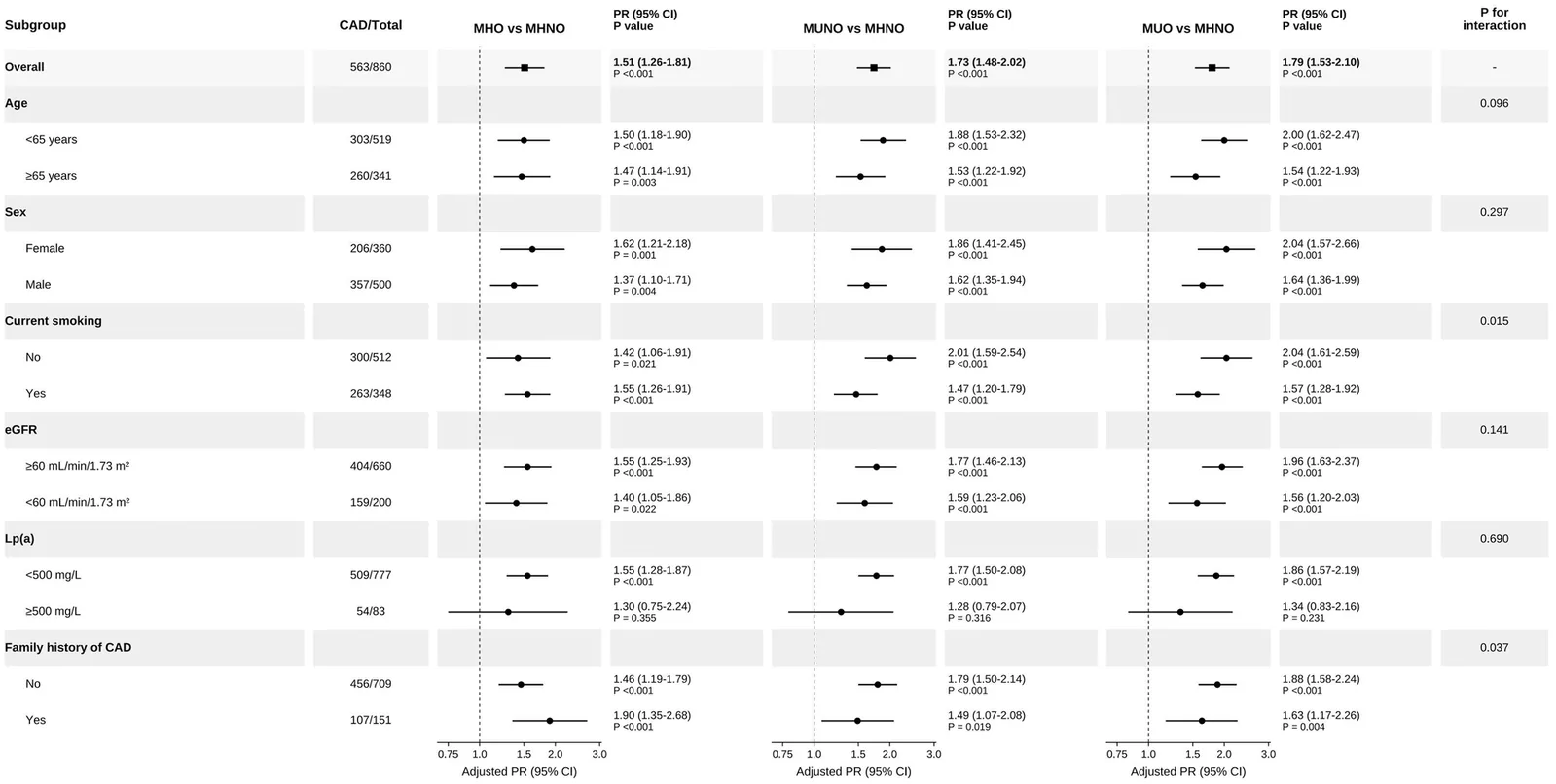
Subgroup analysis of the association between obesity–metabolic phenotypes and CAD prevalence. Adjusted prevalence ratios (PRs) and 95% confidence intervals (CIs) were estimated using modified Poisson regression with HC0 robust variance. MHNO was used as the reference group. Each subgroup model was adjusted for the prespecified covariates except the corresponding stratification variable. CAD, coronary artery disease; CI, confidence interval; eGFR, estimated glomerular filtration rate; Lp(a), lipoprotein(a); MHNO, metabolically healthy non-obese; MHO, metabolically healthy obesity; MUNO, metabolically unhealthy non-obese; MUO, metabolically unhealthy obesity; PR, prevalence ratio.

### Sensitivity analyses

Across the four sensitivity analyses, the direction of the associations between obesity–metabolic phenotypes and CAD prevalence remained unchanged. When severe CAD was defined as a maximum coronary stenosis of ≥70%, the adjusted PRs for MHO, MUNO, and MUO versus MHNO were 2.02 (95% CI, 1.45–2.81), 2.47 (95% CI, 1.84–3.30), and 2.55 (95% CI, 1.89–3.44), respectively. When continuous covariates were modeled using restricted cubic splines, the corresponding PRs were 1.51 (95% CI, 1.26–1.81), 1.72 (95% CI, 1.47–2.01), and 1.79 (95% CI, 1.52–2.09). After excluding patients with ACS, the PRs were 1.69 (95% CI, 1.32–2.16), 2.02 (95% CI, 1.63–2.51), and 2.11 (95% CI, 1.70–2.63), respectively. After additional adjustment for statin use, the corresponding PRs were 1.52 (95% CI, 1.27–1.81), 1.74 (95% CI, 1.49–2.03), and 1.81 (95% CI, 1.55–2.12). The overall association between obesity–metabolic phenotype and CAD prevalence remained statistically significant in all sensitivity analyses (all *P* < 0.001; Table 4).

**Table 4.** Sensitivity analyses of the association between obesity–metabolic phenotypes and CAD prevalence.

Sensitivity analysis	Sample size	CAD cases	MHO vs MHNO, adjusted PR (95% CI)	MUNO vs MHNO, adjusted PR (95% CI)	MUO vs MHNO, adjusted PR (95% CI)	Overall *P* value
Severe CAD (maximum coronary stenosis ≥70%)	860	321	2.02 (1.45–2.81)	2.47 (1.84–3.30)	2.55 (1.89–3.44)	<0.001
Continuous covariates modeled using restricted cubic splines	860	563	1.51 (1.26–1.81)	1.72 (1.47–2.01)	1.79 (1.52–2.09)	<0.001
Excluding patients with ACS	640	364	1.69 (1.32–2.16)	2.02 (1.63–2.51)	2.11 (1.70–2.63)	<0.001
Additional adjustment for statin use	860	563	1.52 (1.27–1.81)	1.74 (1.49–2.03)	1.81 (1.55–2.12)	<0.001

MHNO was used as the reference group. Adjusted PRs and 95% CIs were estimated using modified Poisson regression with HC0 robust variance. The base model included age, sex, current smoking, alcohol use, family history of CAD, Lp(a), LDL-C, and eGFR. In the restricted cubic spline analysis, age, Lp(a), LDL-C, and eGFR were modeled using three knots located at the 10th, 50th, and 90th percentiles. The analysis excluding ACS patients was restricted to participants without ACS. In the statin-adjusted analysis, statin use was additionally included in the base model.

ACS, acute coronary syndrome; CAD, coronary artery disease; CI, confidence interval; eGFR, estimated glomerular filtration rate; LDL-C, low-density lipoprotein cholesterol; Lp(a), lipoprotein(a); MHNO, metabolically healthy non-obese; MHO, metabolically healthy obesity; MUNO, metabolically unhealthy non-obese; MUO, metabolically unhealthy obesity; PR, prevalence ratio.

### Exploratory mediation analysis of hsCRP

An exploratory mediation analysis was performed to assess the potential mediating role of hsCRP in the association between obesity–metabolic phenotypes and CAD prevalence. After adjustment for age, sex, current smoking, alcohol use, family history of CAD, Lp(a), LDL-C, and eGFR, hsCRP levels were higher by 0.27 mg/L (95% CI, 0.10–0.45; *P* = 0.003), 0.93 mg/L (95% CI, 0.72–1.15; *P* < 0.001), and 1.49 mg/L (95% CI, 1.23–1.76; *P* < 0.001) in the MHO, MUNO, and MUO groups, respectively, compared with the MHNO group. The corresponding *P* values for the phenotype-by-hsCRP interaction were 0.851, 0.941, and 0.776.

The average indirect effects for MHO, MUNO, and MUO versus MHNO were 1.94 percentage points (95% CI, 0.25–4.35; *P* = 0.012), 5.93 percentage points (95% CI, 2.15–9.94; *P* = 0.014), and 8.20 percentage points (95% CI, 2.71–13.80; *P* = 0.002), respectively. The corresponding average direct effects were 17.37 percentage points (95% CI, 8.59–26.88), 24.36 percentage points (95% CI, 15.39–33.25), and 26.32 percentage points (95% CI, 16.11–36.13). The proportions mediated were 9.4% (95% CI, 1.4%–25.8%), 19.6% (95% CI, 7.2%–35.3%), and 23.6% (95% CI, 7.9%–43.8%), respectively (Figure 2).

**Figure 2.**
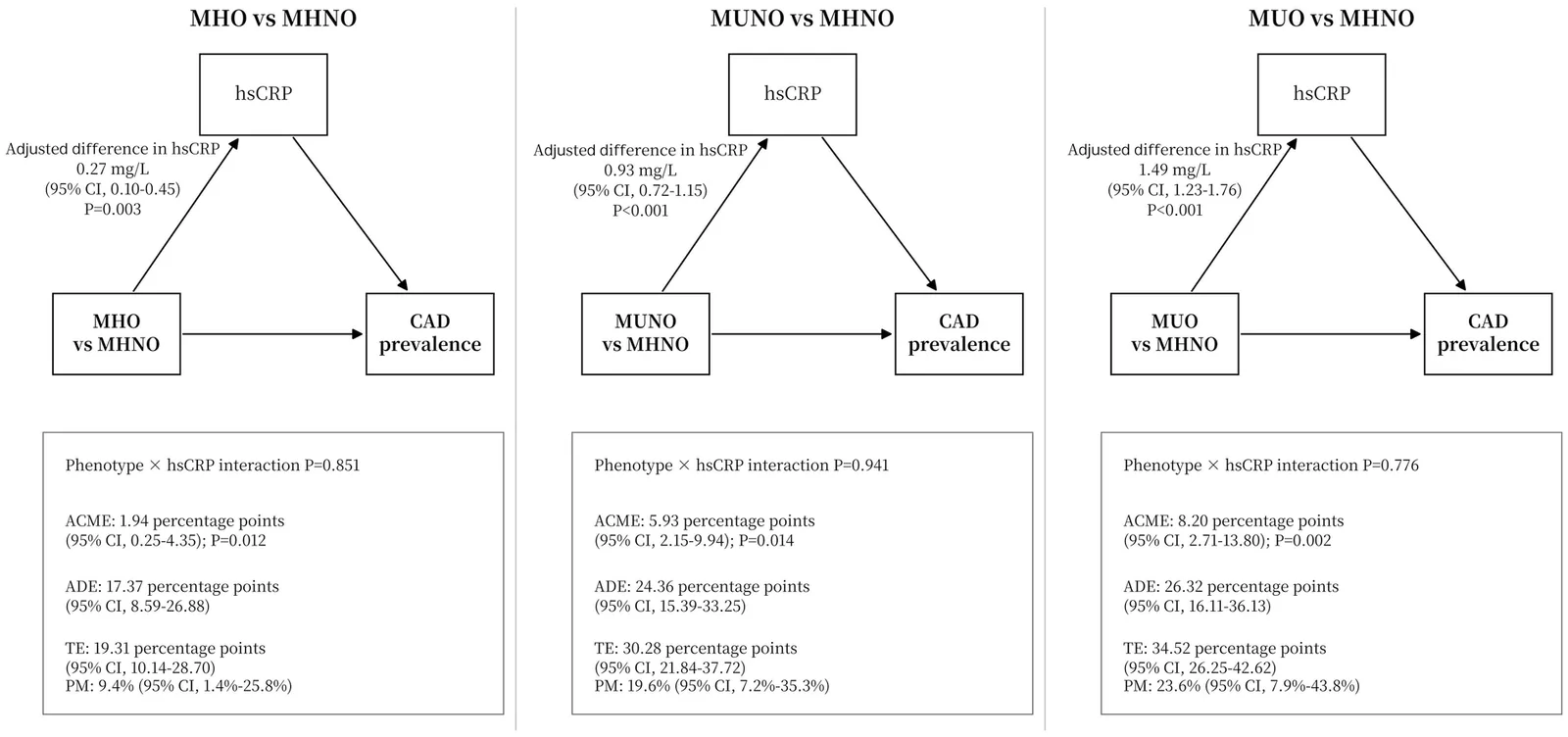
Exploratory mediation analysis of hsCRP in the association between obesity–metabolic phenotypes and CAD prevalence. MHNO was used as the reference group for comparisons with MHO, MUNO, and MUO. Models were adjusted for age, sex, current smoking, alcohol use, family history of CAD, Lp(a), LDL-C, and eGFR, and included the phenotype-by-hsCRP interaction term. The a path represents the adjusted mean difference in hsCRP level associated with each obesity–metabolic phenotype. ACME, ADE, and TE denote the average indirect effect, average direct effect, and total effect, respectively, and are expressed as absolute differences in the predicted probability of CAD in percentage points. PM denotes the proportion mediated. ACME, average indirect effect; ADE, average direct effect; CAD, coronary artery disease; CI, confidence interval; eGFR, estimated glomerular filtration rate; hsCRP, high-sensitivity C-reactive protein; LDL-C, low-density lipoprotein cholesterol; Lp(a), lipoprotein(a); MHNO, metabolically healthy non-obese; MHO, metabolically healthy obesity; MUNO, metabolically unhealthy non-obese; MUO, metabolically unhealthy obesity; PM, proportion mediated; TE, total effect.

## Discussion

In this study, obesity–metabolic phenotypes were differentially associated with CAD prevalence among patients undergoing clinically indicated coronary angiography. Compared with MHNO, MHO, MUNO, and MUO were all associated with a higher prevalence of CAD, with stronger associations observed in the metabolically unhealthy phenotypes. A negative interaction between obesity and metabolic unhealthiness was observed. In parallel, hsCRP levels increased across the phenotypes from MHNO to MUO, and the exploratory mediation analysis showed larger hsCRP-related indirect effects in MUNO and MUO. These findings should be interpreted within the context of this selected clinical population and do not represent CAD risk estimates for the general population.

Whether MHO represents a relatively low-risk cardiovascular phenotype remains controversial. Fauchier et al. found that MHO was associated with some cardiovascular outcomes, although the associations varied across specific endpoints (15). In a Chinese population, Deng et al. reported higher cardiovascular disease incidence in both MHO and MUNO, with the strongest association observed in MUO (16). Evidence from the UK Biobank and the Swedish Cardiopulmonary Bioimage Study (SCAPIS) further suggests that cardiovascular burden is related not only to BMI but also to overall metabolic health and long-term body-weight status (17, 18). The prognostic implications of MHO also depend substantially on how metabolic health is defined (19). In patients undergoing coronary angiography, metabolic syndrome and its components have likewise been associated with angiographically significant CAD (20). Taken together, these findings support the view that MHO should not be regarded as uniformly benign and that its clinical relevance may depend on the accompanying metabolic profile and the cardiovascular outcome being considered.

Our finding that the association with CAD prevalence was stronger for MUNO than for MHO, while being greatest for MUO, further emphasizes the importance of metabolic health. A normal BMI does not exclude adverse fat distribution or cardiometabolic abnormalities. A systematic review showed that individuals with normal-weight obesity may still have substantial abnormalities in glucose and lipid metabolism and other cardiovascular risk factors (21). A recent meta-analysis of abdominal obesity phenotypes likewise found that metabolic unhealthiness, irrespective of abdominal obesity status, was associated with adverse cardiovascular outcomes (22). Obesity–metabolic phenotypes are also dynamic. Persistent metabolic unhealthiness and transition from a metabolically healthy to an unhealthy state have both been associated with subsequent cardiovascular disease (23, 24), suggesting that MHO may represent a transitional rather than a permanently low-risk phenotype (25). In addition, visceral and ectopic adiposity, insulin resistance, and atherogenic dyslipidemia may partly explain discordance between BMI and metabolic health (26–28). Beyond BMI-based obesity classification, the distribution of adipose tissue may provide additional information regarding cardiometabolic risk. Waist-to-hip ratio (WHR), as a simple anthropometric marker, can distinguish predominantly central (android) obesity from lower-body (gynoid) fat distribution, which may have distinct metabolic consequences. Central adiposity is more closely associated with insulin resistance, adverse lipid profiles, systemic inflammation, and cardiovascular risk, whereas gluteofemoral adipose tissue has been suggested to exhibit relatively protective metabolic characteristics (29). Recent imaging-based evidence further indicates that among individuals with similar BMI, android and gynoid obesity may be associated with different cardiac phenotypes, with higher WHR independently related to impaired myocardial deformation in android obesity, while gynoid obesity may show a more favorable myocardial profile (30). These differences in fat distribution may partly contribute to the heterogeneity observed within the MHO phenotype and may explain why BMI alone does not fully capture obesity-related cardiovascular risk. These observations support the simultaneous consideration of obesity and metabolic health when evaluating coronary disease rather than relying on BMI alone.

A notable finding was the negative interaction between obesity and metabolic unhealthiness. This should not be interpreted as a protective effect of obesity because MUO still had the highest prevalence of CAD. Rather, the joint association of obesity and metabolic unhealthiness was smaller than would be expected from a simple combination of their individual associations. Obesity and metabolic unhealthiness share several biological pathways, including adipose tissue dysfunction, ectopic fat accumulation, insulin resistance, and chronic inflammation (31–33). Once these abnormalities are already substantially expressed in metabolically unhealthy individuals, obesity defined by BMI may provide less additional relative information. The high prevalence of CAD in this clinically selected population may also influence interaction estimates on relative and additive scales. Therefore, the observed negative interaction is better interpreted as evidence that obesity and metabolic unhealthiness are not simply additive in their association with CAD. Its reproducibility should be examined in other populations and under alternative definitions of obesity and metabolic health.

The hsCRP findings provide an additional perspective on the heterogeneity of obesity–metabolic phenotypes. Previous population-based studies have linked higher hsCRP levels to clustering of cardiovascular risk factors and greater atherosclerotic burden (34). Upstream inflammatory mediators such as interleukin-6 have also been associated with coronary heart disease and other cardiovascular outcomes (35). Among high-risk patients receiving contemporary lipid-lowering therapy, residual inflammatory risk remains strongly associated with cardiovascular events (36). However, genetic evidence does not consistently support a direct causal role of C-reactive protein (CRP) itself in coronary disease (37). In contrast, the cardiovascular benefits observed in the Canakinumab Anti-inflammatory Thrombosis Outcomes Study (CANTOS) and the Low-Dose Colchicine 2 (LoDoCo2) trial provide stronger evidence that inflammatory pathways contribute to atherosclerotic disease (38, 39). Recent studies have further linked persistent hsCRP elevation to adverse outcomes after percutaneous coronary intervention (40), combined cardiometabolic and inflammatory burden to incident cardiovascular disease (41), and a composite hsCRP–triglyceride-glucose index to vulnerable plaque characteristics on optical coherence tomography (42). Accordingly, the present mediation findings should be interpreted as an hsCRP-related indirect association that may reflect systemic inflammatory pathways, rather than evidence that hsCRP itself is a causal mediator. Importantly, hsCRP was measured during hospitalization for coronary angiography rather than before the development of CAD. Acute coronary events, tissue injury, or other inflammatory conditions at admission may have influenced hsCRP concentrations. Therefore, reverse causation cannot be excluded, and the observed hsCRP-related indirect association should be interpreted cautiously.

Several limitations should be acknowledged. First, the single-center retrospective cross-sectional design precludes determination of temporal sequence among obesity–metabolic phenotypes, hsCRP, and CAD. Causal inference is therefore not possible, and the mediation analysis should be considered exploratory. Second, all participants underwent coronary angiography for clinical indications, and CAD prevalence was relatively high (65.5%). Therefore, this study represents a clinically selected population rather than a community-based sample. Referral and selection bias may have resulted from the inclusion of individuals with symptoms, cardiovascular risk factors, or suspected coronary disease who were referred for angiographic evaluation. Consequently, the observed associations should not be interpreted as population-level estimates, and the generalizability of our findings to the broader population may be limited. Third, obesity was defined using BMI alone, without information on waist circumference, WHR, body fat percentage, or visceral adiposity, which may have resulted in phenotype misclassification. Because BMI does not distinguish fat distribution patterns, individuals with similar BMI values may have substantially different cardiometabolic risk profiles depending on the predominance of central versus lower-body adiposity. Future studies incorporating WHR, imaging-derived visceral fat measurements, or other indicators of adipose tissue distribution are needed to further refine obesity–metabolic phenotypes. Fourth, metabolic health and hsCRP were assessed at a single clinical time point and therefore may not reflect long-term status. Moreover, hsCRP was measured during hospitalization for coronary angiography, when acute conditions such as ACS, tissue injury, or other inflammatory states may influence hsCRP concentrations. The lack of temporal separation between hsCRP measurement and CAD assessment raises the possibility of reverse causation and limits causal interpretation of the exploratory mediation analysis. Fifth, although several clinically relevant factors were considered, residual confounding from diet, physical activity, sleep, socioeconomic factors, and other unmeasured variables cannot be excluded. In addition, some adjusted variables, particularly LDL-C, may represent biological pathways linking metabolic dysfunction to coronary atherosclerosis rather than pure confounders. Therefore, adjustment for these variables may partially attenuate the observed associations, and the estimated effects should be interpreted as adjusted associations rather than total causal effects. Finally, CAD was primarily characterized by maximum coronary stenosis, without detailed assessment of lesion distribution, overall plaque burden, plaque composition, or functional ischemia. Multicenter prospective studies with repeated assessment of metabolic phenotypes and inflammatory status, together with more detailed measures of adiposity and coronary atherosclerosis, are needed to confirm these findings.

## Conclusion

Obesity–metabolic phenotypes were differentially associated with CAD prevalence. MHO, MUNO, and MUO were all associated with higher CAD prevalence than MHNO, with stronger associations observed in metabolically unhealthy phenotypes. Obesity and metabolic unhealthiness showed a negative interaction. Exploratory mediation findings identified an hsCRP-related indirect association, but causal mediation cannot be inferred from this cross-sectional study. These findings support considering metabolic health alongside obesity status when evaluating CAD.

## Data Availability

The original contributions presented in the study are included in the article/Supplementary Material, further inquiries can be directed to the corresponding authors.
